# Complete genome sequences of *Yersinia pestis* 6/69 strain isolated from a bubonic plague patient in Madagascar and its isogenic strain cured of pPCP1

**DOI:** 10.1128/mra.01021-24

**Published:** 2025-02-20

**Authors:** Emelyne Bougit, Guillem Mas Fiol, Pierre Lê-Bury, Charlotte Balière, Valérie Caro, Javier Pizarro-Cerdá, Olivier Dussurget

**Affiliations:** 1 Yersinia Research Unit, Institut Pasteur, Université Paris Cité, Paris, France; 2 WHO Collaborating Research and Reference Centre for Plague FRA-146, Institut Pasteur, Université Paris Cité, Paris, France; 3 Center for Immunology of Viral, Auto‐immune, Hematological and Bacterial Diseases (IMVA‐HB/IDMIT), Université Paris-Saclay, Inserm, CEA, Fontenay‐aux‐Roses, France; 4 Environment and Infectious Risk Unit, Laboratory for Urgent Response to Biological Threats, Institut Pasteur, Université Paris Cité, Paris, France; 5 Yersinia National Reference Laboratory, Institut Pasteur, Université Paris Cité, Paris, France; University of Maryland School of Medicine, Baltimore, Maryland, USA

**Keywords:** *Yersinia pestis*, genome, pPCP1, *pla* gene, plague

## Abstract

We report the complete genome sequences of two valuable strains to investigate plague pathogenesis: (i) *Yersinia pestis* strain 6/69, which was isolated from a bubonic plague patient in Madagascar and contains pCD1, pMT1, and pPCP1 virulence plasmids, and (ii) the 6/69 strain cured of pPCP1.

## ANNOUNCEMENT

*Yersinia pestis* causes plague (1), a life-threatening zoonosis still prevalent in Africa, Asia, and America (2), whose pathogenesis remains to be fully characterized. Here, we report the genome sequences of *Y. pestis* 6/69 and 6/69_pPCP1-_ lacking pPCP1 virulence plasmid. The 6/69 strain was isolated in 1969 at the Institut Pasteur of Madagascar from a patient displaying a bubo in the Fandriana province of Madagascar (3). It belongs to Orientalis biovar, sublineage 1.ORI3 and harbors virulence plasmids pMT1, pCD1, and pPCP1, also known as pFra, pYV, and pPla, respectively (4, 5). Genomic characterization of the 6/69 strain is of interest as it has been used as a reference strain and, along with 6/69_pPCP1-_, is useful for studying virulence genes, in particular, plasminogen activator gene *pla* harbored by pPCP1 (5–11).

To eliminate pPCP1, the 6/69 strain was subcultured 11 times at 4°C on trypticase soy agar with 0.002% hemin. For sequencing, 6/69 and 6/69_pPCP1-_ strains were grown for 16 hours in lysogeny broth at 28°C. DNA was extracted using PureLink Genomic DNA kit (Invitrogen) and quantified using Qubit 2.0 (ThermoFisherScientific). Default parameters were used except where otherwise noted. DNA was sequenced on a MinION-Mk1b using Oxford Nanopore Technologies (ONT) Rapid Barcoding Kit 24 V14 (SQK-RBK14.24), a R10.4.1 flowcell (FLO-MIN114) and MinKNOW/23.04.3 software, generating 80,000 and 33,000-bp length reads for 6/69 and 6/69_pPCP1-_, respectively. Dorado/0.3.0 (https://github.com/nanoporetech/dorado) in sup mode was used for basecalling. In parallel, a library was prepared using Illumina DNA Prep kit and sequenced on a MiSeq system (Illumina), generating 1,359 and 2,158 thousand 2 × 151 bp length reads for 6/69 and 6/69_pPCP1-_, respectively. Hybrid genome assembly based on ONT and Illumina sequencing data was performed using the Easy pipeline (12). ONT reads were filtered with Filtlong/0.2.0 (https://github.com/rrwick/Filtlong) and assembled using Trycycler/0.5.0 (13), Flye/2.9 (14), Raven/1.6.0 (15), and NECAT/0.0.1 (16). Reads were polished using Medaka/1.11.3 (https://github.com/nanoporetech/medaka) and Polypolish/0.1.3 (17). Illumina reads were quality controlled using fastqc/0.12.1 and assembled using fq2dna/23.12 (gitlab.pasteur.fr/GIPhy/*fq2dna*), fqCleanER/23.12 (https://gitlab.pasteur.fr/GIPhy/fqCleanER), and SPAdes/3.15.5 (18). Annotation was performed with Bakta/1.5.0 (19). All reads were provided as FASTQ files.

**Fig 1:**
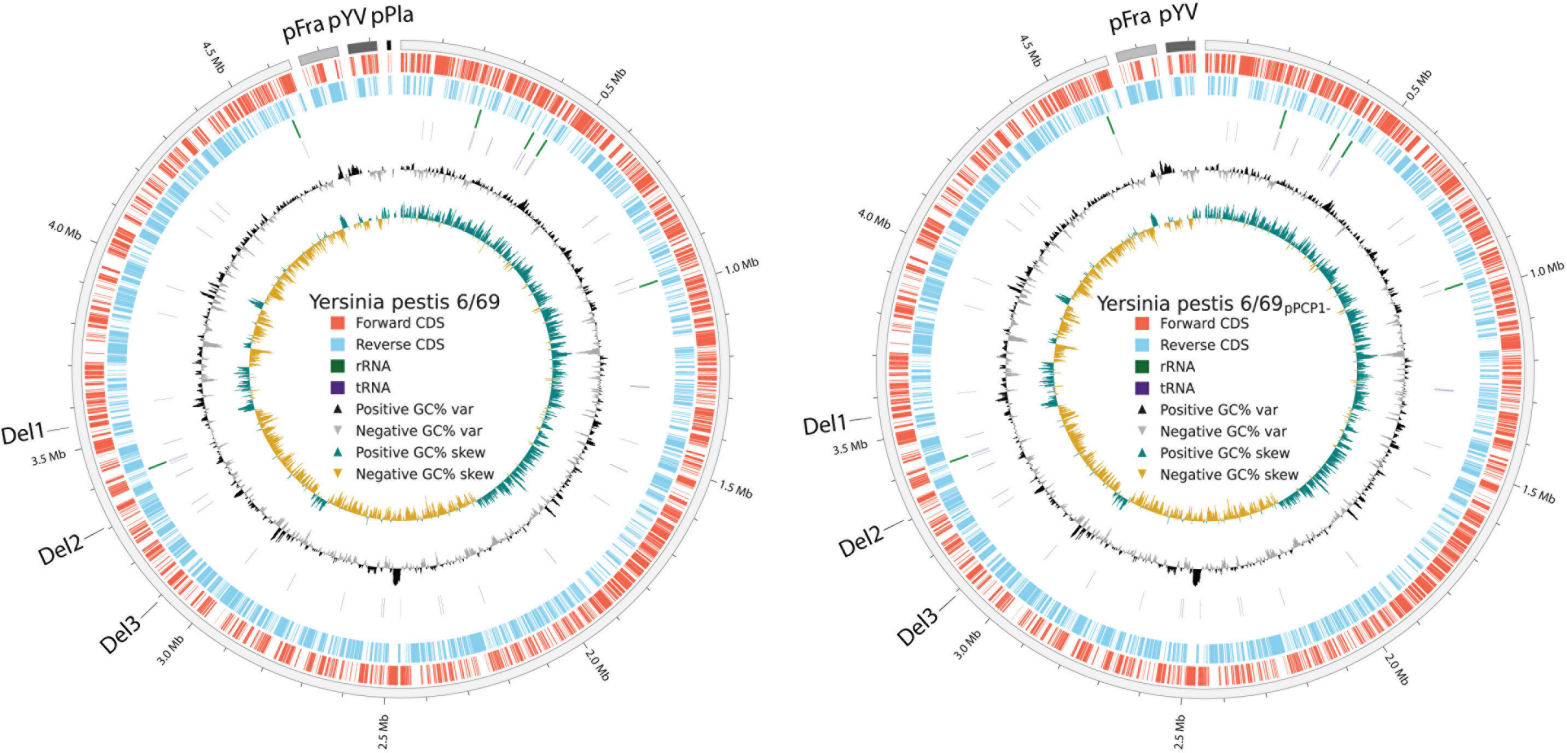
Genome maps of *Yersinia pestis* 6/69 (left) and 6/69_pPCP1-_ (right). Rings from the outside in: scale marks (1), protein-coding genes on the forward strand (2) or reverse strand (3), rRNA genes (4), tRNA genes (5), GC variation (6), and GC skew (7). Positions of the three identified deletions in 6/69 compared to CO92 are displayed (Del1, Del2, and Del3). Created using Circos.

The 6/69 strain possesses a 4.8 Mb circular chromosome and the three plasmids found in the CO92 reference strain (1) (Table 1; Fig. 1). The 6/69 and CO92 strains have an average nucleotide identity >99%. They differ by 52 chromosomal single nucleotide polymorphisms and 3 deletions of 1,510, 10,344, and 3,480 bp starting at positions 657344, 1310775, and 1534290 of CO92 chromosome (GCF_000009065.1). The absence of pPCP1 is the only difference between 6/69_pPCP1-_ and 6/69 strains, highlighting their relevance as tools to study the role of pPCP1 in pathogenesis.

**TABLE 1:** General features of *Y. pestis* 6/69 and 6/69_pPCP1-_

	6/69					6/69_pPCP1-_			
**Parameters**	**Chromosome**	**pMT1**	**pCD1**	**pPCP1**	**Whole genome**	**Chromosome**	**pMT1**	**pCD1**	**Whole genome**
GenBank accession no.	CP166966.1	CP166967.1	CP166968.1	CP166969.1		CP166963.1	CP166964.1	CP166965.1	
No. of circular contigs	1	1	1	1	4	1	1	1	3
Size (bp)	4,649,807	96,210	70,300	9,610	4,825,927	4,649,807	96,210	70,300	4,816,317
G + C content (%)	47.6	50.2	44.8	45.3	47.6	47.6	50.2	44.8	47.6
No. of annotated protein-coding genes	4,097	99	90	10	4,296	4,097	99	90	4,286
No. of pseudogenes	8	2	0	0	10	8	2	0	10
No. of rRNAs	19	0	0	0	19	19	0	0	19
No. of tRNAs	71	0	0	0	71	71	0	0	71
No. of ncRNAs	183	1	2	2	188	183	1	2	186
CRISPR array	4	0	0	0	4	4	0	0	4
Total no. of reads	80,656 (ONT) 1,359,952 paired reads (Illumina)					33,370 (ONT) 2,158,311 paired reads (Illumina)			
ONT *N*_50_ length	14,027					14,960			
Total no. of bases	624,569,231 (ONT) 203,992,800 (Illumina)					249,181,216 (ONT) 321,207,344 (Illumina)			
Sequencing depth (×)	129 (ONT) 42 (Illumina)					51 (ONT) 66 (Illumina)			

## Data Availability

Genome sequences of *Y. pestis* 6/69 (IP304) and 6/69_pPCP1-_ (IP343R) were submitted to NCBI under BioProject ID PRJNA1128573 and BioSample numbers SAMN42052713 and SAMN42053676. Assembled genomes are available in GenBank under numbers GCA_041410195.1 and GCA_041409745.1. Illumina and ONT data can be accessed on Sequencing Read Archive numbers SRR29554089, SRR29554088 and SRR29554145, SRR29554144, respectively. Furthermore, sequences are available on Yersiniomics (20), providing structural and functional properties of gene products.
